# Case Report: Acute hallucinations and delusions following an argument, attributed to temporal lobe hypoperfusion

**DOI:** 10.3389/fpsyt.2026.1780803

**Published:** 2026-03-09

**Authors:** Shu Xie, Yao Yang, Zhibo Ouyang, Yun Zhang, Jian Shi

**Affiliations:** Department of Psychosomatic Medicine and Sleep Medicine Center, Mianyang Central Hospital, School of Medicine, University of Electronic Science and Technology of China, Mianyang, China

**Keywords:** delusions, depression, hallucinations, perfusion imaging, temporal lobe

## Abstract

**Purpose:**

Hallucinations and paranoid delusions are common psychiatric symptoms that can lead to dangerous behaviors such as self-harm and aggression. The temporal lobe is a key region for emotional processing, and its impairment can cause symptoms such as anxiety, depression, cognitive deficits, hallucinations, and delusions. The main purpose of this article is to discuss the effect of improved hypoperfusion on one patient’s psychiatric symptoms.

**Patient and Methods:**

A 61-year-old male with untreated hypertension was transferred from the neurology department after developing hallucinations and persecutory delusions following a domestic argument. Perfusion-weighted magnetic resonance imaging (PWI) showed hypoperfusion in the temporal lobe.

**Results:**

Fluid therapy was immediately initiated to improve temporal lobe perfusion, and a low dose of anti-psychotic medication was maintained for a short time. After eight days of fluid therapy, the patient’s hallucinations and delusions had substantially improved. A subsequent PWI revealed a marked improvement in both the relative cerebral blood flow and relative cerebral blood volume in the left temporal lobe. After discharge, anti-psychotic medications were stopped after one month. No recurrence of psychiatric symptoms was observed during a subsequent five-year monitoring period.

**Conclusion:**

The initial appearance of psychiatric symptoms requires vigilance for an underlying organic cause. Emotional agitation can cause insufficient cerebral perfusion in stenotic vessels, and hypoperfusion of the left temporal lobe may present as hallucinations or delusions. PWI and similar methods can help to differentiate organic brain disease from primary psychiatric disorders, and early restoration of cerebral blood flow often correlates with a good prognosis.

## 1 Introduction

Acute hallucinations and delusions are common clinical syndromes in psychiatric and neurological emergencies, and have complex etiologies. Differential diagnoses include schizophrenia, affective psychosis, substance abuse, and a range of organic brain diseases. Traditionally, these symptoms have frequently been attributed to dysfunction in the brain’s neurotransmitter systems. However, with advances in neuroimaging techniques, increasing evidence suggests that structural or functional damage in specific brain regions can directly lead to complex psychotic symptoms (1–3). Among these regions, the temporal lobe, particularly the left temporal lobe, has increasingly attracted attention owing to its association with language, auditory processing, memory, and emotional integration.

The temporal lobe is not a functionally homogeneous brain region. In individuals with a dominant hemisphere, the left temporal lobe is responsible for essential specialized functions. Its upper part, particularly the superior temporal gyrus, encompasses the primary auditory cortex and the classic Wernicke’s area, which are responsible for receiving auditory information and language comprehension (4). The hippocampus and amygdala, integral components of the deeper medial temporal lobe, are pivotal in the formation of declarative memories and the modulation of emotional responses. Furthermore, the temporal lobe is intricately connected with the frontal lobe, parietal lobe, and limbic system via a dense network of fibers, including the arcuate fasciculus and uncinate fasciculus. These connections are central in facilitating complex cognitive functions such as social cognition, reality monitoring, and the sense of self. Therefore, the functional integrity of the left temporal lobe is essential for maintaining normal thought content, perceptual experience, and reality-testing ability (5).

Insufficient perfusion (i.e., a substantial reduction in local cerebral blood flow [CBF]) can lead to neuronal metabolic crisis and functional suppression, and its effects possibly precede the appearance of structural lesions (4, 6). When insufficient perfusion occurs in the left temporal lobe, the function of the above-mentioned key networks may undergo acute breakdown. Ischemia in the language comprehension area can cause sensory aphasia and may present as delusions such as the impression of being watched or talked about. Dysfunction in the auditory association cortex may trigger complex auditory hallucinations. Involvement of the limbic system can directly lead to emotional blunting, fear, or emotional dysregulation, providing an emotional basis for delusions. This neuropsychiatric syndrome highlights the direct link between brain structure and mental phenomena (7, 8). This article reports a case that primarily presented with acute hallucinations and delusions. The diagnosis was focal hypoperfusion in the left temporal lobe based on computed tomography angiography and perfusion-weighted magnetic resonance imaging (MRI) (PWI). Common organic causes such as tumors and infections were ruled out. The report uses this case to elucidate the diverse roles of left temporal lobe subregions, including language processing, auditory comprehension, and memory retention, and their potential pathological links to psychotic symptoms. It highlights the importance of examining non-psychiatric factors, particularly those related to vascular health, and hypoperfusion-related causes in the differential diagnosis of acute psychiatric symptoms, as well as exploring the importance of functional neuroimaging in demonstrating the neural basis of psychiatric symptoms (9, 10). Through this case, we hope to deepen clinicians’ understanding of “organic psychosis” and promote more precise diagnostic and treatment strategies.

## 2 Case presentation

### 2.1 Patient information

The patient was a 61-year-old heterosexual male, married with children. He had no religious beliefs or family history of mental illness. He had hypertension but did not take medication. The ethics committee of Mianyang Central Hospital approved this study, and all data have been anonymized. The participant provided his written informed consent.

### 2.2 Chief complaint

The patient’s chief complaints were visual hallucinations and delusions of persecution, which had persisted for 5 days. He was taken to hospital by his family.

### 2.3 History of present illness

The patient presented with symptoms of visual hallucinations, persecution delusions, and dysphoria after an argument with his wife. The main manifestations were hallucinations of friends from his younger days scolding him, and feelings that the environment around him was strange and that his family and friends were unfriendly and could harm him through poisoning or other specific means. The patient had also insulted his family and had impulsive speech.

The patient was admitted to the neurology department. A neurological consultation included a head MRI that revealed scattered punctuate ischemic lesions but no other abnormalities, and no specific treatment was needed. After consultation in the psychosomatic medicine department, he was diagnosed with delusional syndrome, and transferred to the psychosomatic medicine department for further treatment.

### 2.4 Previous medical history

The patient had been diagnosed with hypertension two years ago but no specific treatment had been administered. He reported no diagnoses of diabetes, hyperlipidemia, coronary heart disease, stroke or other diseases, and no history of any other major physical diseases or of food or drug allergies.

### 2.5 Family members and personal growth history

The patient’s parents had a harmonious relationship, and he had experienced no major psychological traumas. The patient was a farmer and got along well with his wife and neighbors. He had one child. Overall, he had a harmonious relationship with his wife. He had smoked for 40 years, an average of 15 cigarettes a day, but reported no use of alcohol or other psychoactive substances.

## 3 Assessment

### 3.1 Psychological assessment

The patient’s Hamilton Anxiety Scale score (32) indicated moderate to severe anxiety. His Hamilton Depression Scale score (22) indicated moderate depression.

### 3.2 Mental status and neurological examination

Appearance and Behavior: The patient was dressed appropriately, nervous, passive in contact, and unwilling to reveal his thoughts in detail. Mood and Affect: He exhibited low mood and depression, with obvious anxiety symptoms. He felt that the environment was not safe, and his emotional response was not coordinated with the environment. Thought Content: The patient exhibited symptoms of paranoid delusions, including auditory and visual hallucinations (in which he believed he was communicating through the air) and delusions of persecution and reference (in which he was convinced that his wife and those close to him were plotting to murder him).

Neurological examination: The patient exhibited full consciousness, with hallucinations but coherent speech. Cranial nerve examination revealed symmetrical bilateral frontal wrinkles and nasolabial folds, with no ptosis. The pupils were equal in size and round, measuring approximately 3 mm in diameter, and demonstrated sensitive light reflexes. Bilateral hearing was normal, and tongue protrusion was centrally positioned. Muscle strength: The patient had Grade 5 muscle strength in all four limbs with normal muscle tone. Coordination: The finger-to-nose test and heel-knee-shin test were performed with precision. Sensory system: There was symmetrical presence of pinprick sensation and tuning fork vibration in all four limbs. Reflexes: There was symmetrical elicitation of biceps brachii, patellar, and Achilles tendon reflexes (++), with no pathological signs. Meningeal irritation signs were negative.

### 3.3 Laboratory tests and imaging exam

**Figure 1:**
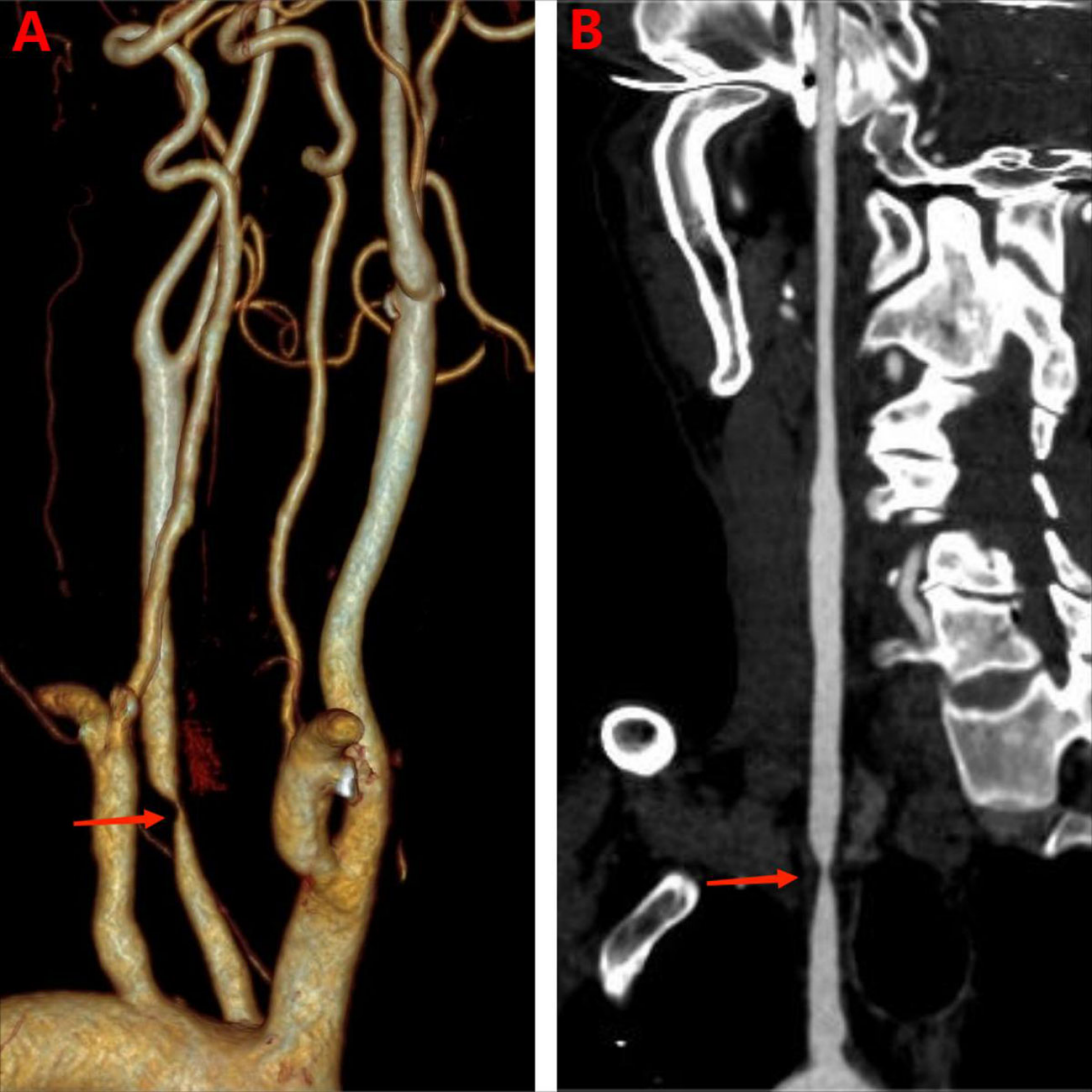
CTA imaging. **(A)** Three-dimensional reconstruction images of the patient before treatment. The arrow indicates severe stenosis of the left internal carotid artery. **(B)** Pre-treatment tomographic images of the patient. The arrows indicate severe stenosis of the left internal carotid artery.

**Figure 2:**
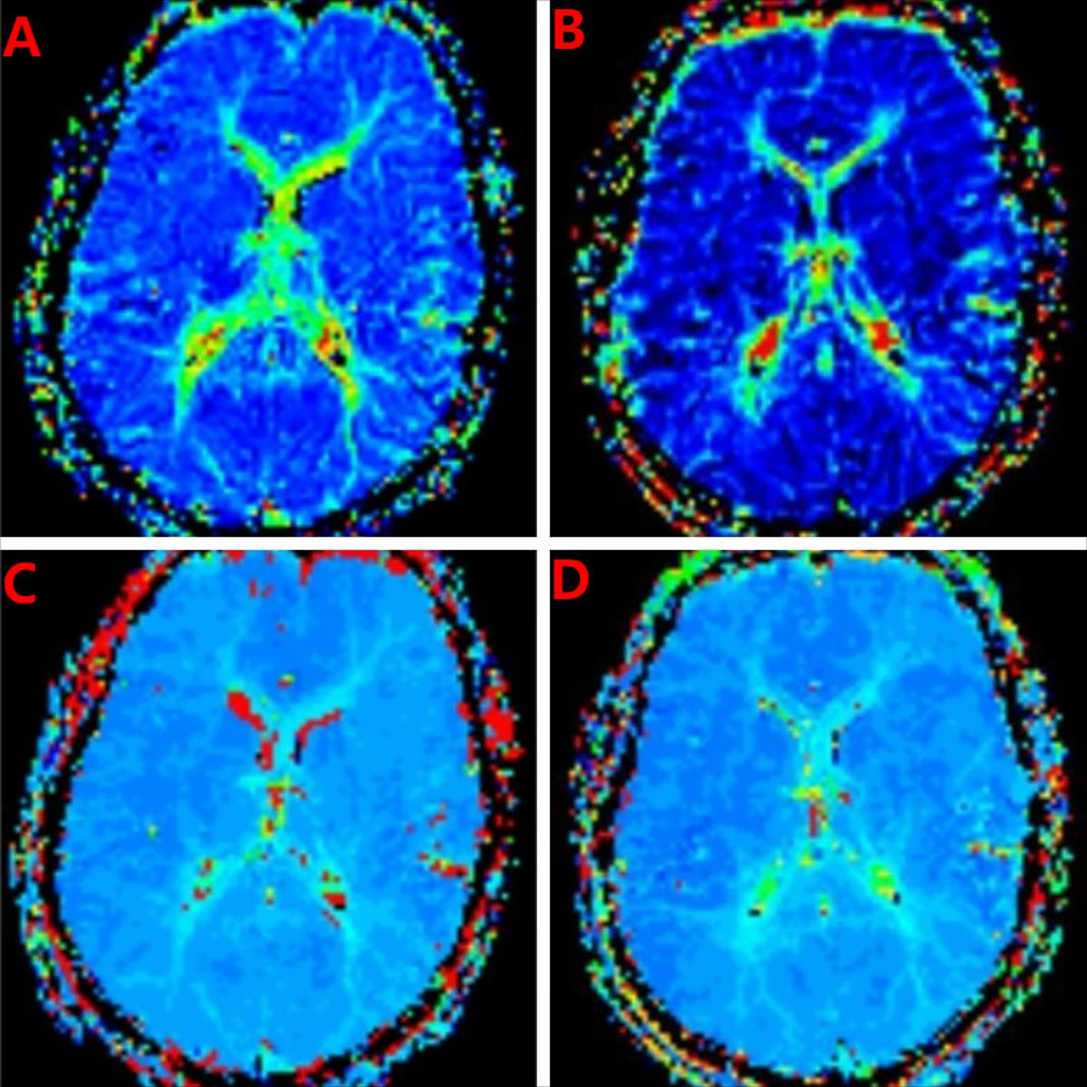
PWI imaging. **(A)** Before treatment, the left temporal lobe showed patchy prolongation of mean transit time (MTT). **(B)** The mean transit time (MTT) of the left temporal lobe showed improvement post-treatment, as evidenced by MRI studies. **(C)** Before treatment, patchy areas with prolonged time to peak (TTP) were observed in the left temporal lobe. **(D)** Following treatment, a significant improvement was observed in the time to peak (TTP) of the left temporal lobe, as indicated by MRI assessments.

We performed the necessary physical examinations to rule out organic disease. (**Table 1**). The CTA showed that low density at the proximal segment of the left internal carotid artery without enlargement, and severe luminal stenosis (**Figures 1A, B**). We were concerned about mental disorders caused by cerebral hypoperfusion and performed a cranial PWI. The PWI showed striped areas in the left temporal lobe with prolonged mean transit time (MTT) and time to peak (TTP) (**Figures 2A, C**), and decreased relative CBF (rCBF) and relative cerebral blood volume (rCBV), suggesting reduced blood flow in the left temporal lobe. After eight days of fluid therapy, the patient’s MTT and TTP showed substantial improvement.

**Table 1:** Laboratory tests and imaging exam results.

Inspection item	Inspection result
Routine blood tests	normal
liver and kidney function	normal
thyroid function	normal
cortisol	normal
syphilis	normal
HIV	normal
hepatitis B	normal
complete immunological tests	normal
tumor markers	normal
Video electroencephalogram (EEG)	normal
cerebrospinal fluid pressure	85 mmH2O
cerebrospinal fluid white blood cells, red blood cells, protein, Acid-fast staining, bacterial and fungal smears	normal
Serum and cerebrospinal fluid autoimmune encephalitis antibodies	Negative(NMDA IgG, AMPA1 IgG, AMPA2 IgG, LGI1 IgG, CASPR2 IgG, GABA B IgG)
CTA	low density at the proximal segment of the left internal carotid artery without enlargement, and severe luminal stenosis (Figures 1A, B)
PWI	the left temporal lobe with prolonged Mean Transit Time (MTT) and Time to Peak (TTP) (Figures 2A, C), and decreased relative Cerebral Blood Flow (rCBF) and relative Cerebral Blood Volume (rCBV)
PWI (after 8 days of treatment)	the patient’s MTT and TTP showed significant improvement (Figures 2B, D)

NMDA IgG, Anti - N - methyl - D - aspartate receptor immunoglobulin G; AMPA1 IgG, Anti - α - amino - 3 - hydroxy - 5 - methyl - 4 - isoxazolepropionic acid receptor subunit 1 immunoglobulin G; AMPA2 IgG, Anti - α - amino - 3 - hydroxy - 5 - methyl - 4 - isoxazolepropionic acid receptor subunit 2 immunoglobulin G; LGI1 IgG, Anti - Leucine - rich glioma - inactivated 1 immunoglobulin G; CASPR2 IgG, Anti - Contactin - associated protein - like 2 immunoglobulin G; GABA B IgG, Anti - γ - aminobutyric acid type B receptor immunoglobulin G; CTA, Computed Tomography Angiography; PWI, Perfusion-Weighted MRI.

### 3.4 Diagnostic assessment

This was based on the Diagnostic and Statistical Manual of Mental Disorders, Fifth Edition (DSM-5), criteria for psychotic disorder due to another medical condition (temporal lobe hypoperfusion).

### 3.5 Case conceptualization

The patient had no previous history of mental illness. His experience of hallucinations and persecutory delusions was sudden, accompanied by considerable anxiety, agitation, and behavioral disturbances. These symptoms are difficult to explain given the patient’s personality traits and emotional evolution, and therefore more likely reflect pathological changes.

## 4 Therapeutic intervention and follow-up

The patient was administered 1500 ml of saline for fluid therapy, along with 500 ml of low-molecular-weight dextrorotatory glucose amino acid injection once daily for 8 days for volume expansion therapy. Follow-up PWI still showed reduced perfusion in the left temporal lobe, but there was improvement compared to before. The patient’s psychiatric symptoms were substantially relieved. He received olanzapine 5 mg qn for 14 days (total treatment duration of 14 days); at discharge, olanzapine was adjusted to 2.5 mg qn. The patient stopped the medication on his own after 1 month. After one year of follow-up, he remained free of psychotic symptoms. At five years, the patient continued to be symptom-free and was leading a normal life.

## 5 Discussion

This report describes a case of acute hallucinations and delusions for which PWI identified focal hypoperfusion in the left temporal lobe as the core etiology. These sudden-onset symptoms of hallucinations and delusions strongly suggest an organic disease, the mechanism of which may involve neural network dysfunction caused by ischemia in functional brain regions. Here, we further analyze the function of the left temporal lobe and the neurobiological mechanism of hypoperfusion-induced psychiatric symptoms, and consider key differential diagnostic approaches and the associated clinical implications.

In clinical practice, the differential diagnosis of acute mental disorder is undoubtedly important, playing a pivotal role in accurate disease assessment and subsequent development of scientifically validated treatment plans. First, organic etiologies must be prioritized, as pathological changes or functional abnormalities in bodily organs can frequently trigger acute mental disorders, necessitating comprehensive and meticulous investigation of such causes. Second, given the complex nature of substance use and its diverse effects on mental states, the possibility of substance-induced mental disorders must be thoroughly evaluated using multidimensional analysis and differentiation. Finally, primary mental disorders should also be considered. Primary mental disorders are characterized by various types and clinical manifestations; schizophrenia is a typical and common example. In this case, the patient exhibited clear organic etiology, and substantial symptom relief was achieved following treatment of the underlying organic cause.

Hallucinations are associated with many neurological disorders and may be an early predictive indicator (11, 12). Abnormalities in connectivity and brain lateralization may be related to the etiopathogenesis of schizophrenia and auditory verbal hallucinations (4). Hallucinations likely arise from transient abnormal activation of sensory cortices, including the superior temporal gyrus, combined with hyperactivity in the default mode network. This network, which supports internal thought and self-referential processing, involves regions such as the medial prefrontal cortex and posterior cingulate cortex (13). Reduced perfusion likely first impairs temporal lobe processing of external auditory information, causing excessively active internal thoughts to be misperceived as external sounds. Delusions are more closely linked to elevated intrinsic activity and connectivity within default mode network regions involved in belief formation and social cognition, such as the medial prefrontal cortex. Dysfunction in the left temporal lobe, particularly areas governing language comprehension, may disrupt an individual’s ability to reality-test internal thoughts against external inputs, thereby facilitating the formation of pathological beliefs (14).

The left temporal lobe, particularly in the dominant hemisphere, serves as a high-level hub for integrating auditory, language, memory, and emotional functions (15). Acute psychiatric symptoms resulting from temporal lobe hypoperfusion likely reflect not a single brain region becoming “silent” but rather “disconnection” and “dysfunction” in specific functional networks (16), leading to collapse of the prediction and self-recognition system. A recent study offers a key insight into the neural basis of hallucinations. It demonstrates that in healthy individuals, the left middle and superior temporal gyrus exhibit normal sensory attenuation during self-initiated actions, a mechanism that allows the brain to distinguish self-generated from external events. In schizophrenia patients, this region displays a dual functional impairment: sensory attenuation is absent during active conditions and baseline activation is reduced under passive conditions (17). This suggests that both the “feedforward prediction” and “sensory feedback integration” mechanisms mediated by this region are impaired. In the current case, hypoperfusion in the left temporal lobe can be considered an acute physical suppression of this region’s function. Consequently, the patient lost the ability to differentiate intrinsic thoughts, which arise from internal associative networks, from external perceptions originating from sensory input. Because this intrinsic cognitive activity could not be correctly “tagged” as self-generated, its vivid mental imagery was experienced as an externally sourced, real “hallucinatory” perception (18).

A more comprehensive integrative model posits that psychotic experiences, such as hallucinations and delusions, arise from a functional reorganization of the brain’s intrinsic activity patterns (14). Specifically, sensory systems, such as the auditory cortex located in the temporal lobe, may exhibit baseline functional deficits or demonstrate abnormal processing of incoming stimuli. The intrinsic activity of the “default mode network,” which is related to internal thought and self-referential processing, and the associative cortices (including the temporoparietal junction and medial prefrontal cortex), is excessively enhanced with increased connectivity. In this case, hypoperfusion in the left temporal lobe directly caused a “functional deficit” in local sensory and language-associated cortices. This deficit may interact with functional compensation or decompensation across the whole-brain network, leading the brain to overly rely on internal associative patterns during information processing and minimizing adaptive changes to a variable environment. Ultimately, this imbalance between internal and external systems causes highly vivid, emotionally colored internal images (e.g., feelings of being watched) to be experienced as undeniable external reality, resulting in delusions (18, 19).

Re-examining organic and primary psychiatric disorders constitutes the most important differential diagnosis. For first-onset, acute, and particularly psychotic symptoms accompanied by any neurological soft signs or vascular risk factors, a systematic evaluation for organic causes is essential. This case demonstrates that for any new-onset, acute, or subacute psychotic symptoms, organic causes must be prioritized in the differential diagnosis. Unlike patients with delirium and other acute brain syndromes, this patient remained fully conscious with a localized lesion, which further illustrates that focal brain dysfunction can directly mimic the manifestations of primary psychiatric disorders (20–22).

. After volume expansion and fluid supplementation, the psychiatric symptoms entirely disappeared. This successful case not only confirms the identification of the pathological mechanism in this case but also highlights the unique value of PWI in identifying reversible ischemic lesions (such as the ischemic penumbra) whose abnormalities may appear earlier than structural infarction (diffusion-weighted imaging [DWI] hyperintensity), providing a window for early intervention (23, 24).

In this case, the patient’s DWI was normal, whereas PWI indicated stripe-shaped prolonged TTP and MTT in the left temporal lobe with reduced rCBF and rCBV, suggesting reduced left temporal lobe perfusion. Therefore, PWI can identify hidden ischemic causes. Although conventional structural MRI may not show a clear infarct, and DWI may be normal or show only minor abnormalities, PWI can directly demonstrate insufficient blood perfusion in the left temporal lobe. This provides a direct, objective organic cause for acute psychiatric symptoms, shifting the diagnosis from a primary psychiatric disorder to vascular-related brain dysfunction. By comparing the low-perfusion area shown on PWI with the infarct core area shown on DWI, doctors can visually identify the “mismatch area” between the two. This area, known as the ischemic penumbra, is characterized by impaired neuronal function but intact structure, rendering it an important target for immediate reperfusion therapy. The focal hypoperfusion of the left temporal lobe revealed by PWI serves as a key link between vascular lesions and psychiatric symptoms (25, 26). This finding occurred earlier than, or independently of, the potential structural infarction DWI evidence, suggesting that the patient’s psychiatric symptoms stemmed from functional ischemic suppression of the left temporal lobe network rather than permanent infarct damage. This perfectly illustrates the concept of the “ischemic penumbra” in neuropsychiatric symptoms and provides solid imaging evidence for a treatment strategy aimed at reversing psychiatric symptoms through urgent vascular intervention to restore perfusion.

From symptomatic treatment to etiological intervention, the treatment strategy for this type of case should follow the principle of addressing both symptoms and root causes: symptomatic treatment during the acute phase is necessary, and for severe psychotic symptoms, the short-term use of low-dose, high-potency antipsychotic drugs to control agitation, hallucinations, and delusions is required, while closely monitoring adverse reactions, especially in patients who may have underlying cerebrovascular disease. The duration of drug treatment for psychiatric symptoms has always been a controversial issue. Primary psychiatric disorders generally require longer treatment. However, in this case, the patient’s psychiatric symptoms were induced by an organic disease, so we used psychiatric medications for a relatively short period (27). The essence of fundamental etiological treatment is in improving the hypoperfusion state of the left temporal lobe. This requires multidisciplinary collaboration between neurology, psychiatry, interventional radiology, and other departments. It is essential to quickly determine the etiology of cerebral hypoperfusion, as it can be caused by conditions such as large artery stenosis, cardioembolic events, or small vessel disease. Treatment options may include antiplatelet/anticoagulant therapy, intensive statin therapy, or, when there is clear large vessel stenosis and indications are met, endovascular intervention (such as stent placement) for revascularization (28–30). Although our patient refused vascular intervention, we were still able to improve the prognosis using volume expansion and fluid therapy. The prognosis of psychiatric symptoms resulting from insufficient CBF is contingent upon the reversibility of the ischemic factors and the promptness of therapeutic intervention. If perfusion can be restored early, there is a possibility of complete recovery of neurological function, including psychiatric symptoms. During the rehabilitation period, neuropsychological assessment should be conducted, and rehabilitation training should target any potential mild cognitive or language deficits. Long-term management should focus on secondary prevention of cerebrovascular disease.

## 6 Conclusion

This case has several important implications. It not only deepens theoretical understanding but also demonstrates core problems, provides decision-making references, promotes interdisciplinary integration, and fosters the ability to analyze and solve problems. At the mechanistic level, it serves as a vivid “natural experiment,” confirming the critical role of the left temporal lobe (particularly the networks involved in sensory integration, self-recognition, and language processing) in maintaining normal reality perception. Dysfunction in this area can directly lead to psychotic symptoms by mimicking the mechanisms of left temporal lobe disturbances. At the diagnostic level, this case strongly suggests the need for a systematic neuroimaging evaluation, including high-resolution MRI and PWI, for patients experiencing a first psychotic episode, especially those with acute onset or atypical features (e.g., older age, cerebrovascular risk factors, or subtle neurological signs). This helps to prevent misdiagnosis of organic brain disease as a primary psychiatric disorder, thereby avoiding delays in fundamental treatment. At the therapeutic level, this case suggests that managing such patients requires an integrated approach. While controlling psychiatric symptoms, neurologists should actively search for and address potential cerebrovascular- or perfusion-related causes. Successful revascularization can lead to substantial improvement or even complete remission of psychiatric symptoms, providing a hopeful counterexample to the traditional view that “psychiatric symptoms are irreversible.” After reviewing the relevant literature, we found no reports of hypoperfusion solely caused by carotid artery stenosis leading to hallucinations and delusions.

In summary, this case advances our understanding of the “brain–psyche” connection, showing that specific, localized cerebrovascular changes are sufficient to trigger complex psychiatric syndromes. Clinically, maintaining high vigilance for potential organic causes underlying psychiatric symptoms and making full use of advanced neuroimaging tools are essential for making an accurate diagnosis and providing effective treatment.

## Data Availability

The original contributions presented in the study are included in the article/supplementary material. Further inquiries can be directed to the corresponding author.
